# The Dowager Lady Roberts

**Published:** 1921-01-01

**Authors:** 


### The Dowager Lady Roberts.

Few women have succeeded in leaving a more permanent impress on their times than the widow, recently passed away, of the great Lord Roberts. We are privileged to transcribe from a letter she wrote in 1891 to the late Sir Henry Burdett an account of the rise and progress of the scheme she initiated for extending the benefits of trained nursing in India. She writes:—

"The objects of my fund are threefold: first, to supply homes in the hills for the lady nurses working in the military hospitals in India, to which they can go for change and rest when worn out by working in the trying climate of the plains, thus preventing their having to take leave to England or remain on in the plains until their health is completely broken down; secondly, in connection with these homes, to provide officers' hospitals where officers who have not homes of their own can be sent in case of illness or when recovering from illness, for many a poor young officer (after a hard fight for life in the plains) has succumbed on being sent to the hills to complete his recovery to the bad, improper food of an Indian hotel or club and from the want of skilled nursing care which is provided for him in my hospitals or convalescent homes; thirdly, to provide an auxiliary staff of lady nurses to work in those military hospitals to which the Government nurses have not been supplied. At Murree, which is the hill station for the Punjaub and North-West of India, is established my largest home. . . . A smaller home has been built at Kasauli, and one at Wellington, in the Madras Presidency. And last year I built a home at Quetta, which has now become a very large cantonment for European troops. That the Government have recognised the necessity for these officers' hospitals is shown by the fact that the patients are allowed to be attended by the depôt surgeons and that medicines are supplied gratis from the Government dispensary. The officers are charged—for those not above the rank of captain five rupees a day, for those above that rank six rupees. The fees charged cover their actual expenses, but the nurses' salaries, building expenses, hospital necessaries, etc., come out of my fund. The auxiliary staff of nurses are for the soldiers. If officers are nursed by them they pay a small sum, which goes to my fund. After paying all expenses to date I have invested Rs.114,000, which brings me in about £500 a year. While I am in India I am so well supported by the Army at large that I can calculate on pretty well paying the current expenses, but I have to work very hard to do so. My great anxiety is to leave enough money invested when I leave India to prevent the work collapsing on my departure."

How well Lady Roberts succeeded in her aim of so laying the foundation that the work should not collapse is matter of history. After the Boer war the whole system of army nursing was thoroughly reorganised. Lady Roberts was offered, and accepted, a seat on the board of Queen Alexandra's Imperial Service, where her knowledge of Indian requirements was of great value. It was her privilege to demonstrate the possibility of introducing lady nurses into India, and in this sense she truly ranks among the chosen band of the pioneers.

